# Novel Biomarkers, Diagnostic and Therapeutic Approach in Rheumatoid Arthritis Interstitial Lung Disease—A Narrative Review

**DOI:** 10.3390/biomedicines10061367

**Published:** 2022-06-09

**Authors:** Alesandra Florescu, Florin Liviu Gherghina, Anca Emanuela Mușetescu, Vlad Pădureanu, Anca Roșu, Mirela Marinela Florescu, Cristina Criveanu, Lucian-Mihai Florescu, Anca Bobircă

**Affiliations:** 1 Department of Rheumatology, University of Medicine and Pharmacy of Craiova, 200349 Craiova, Romania; alesandra.florescu@umfcv.ro (A.F.); anca.rosu@umfcv.ro (A.R.); cristina.criveanu@umfcv.ro (C.C.); 2 Department of Physical Medicine and Rehabilitation, University of Medicine and Pharmacy of Craiova, 200349 Craiova, Romania; florin.gherghina@umfcv.ro; 3 Department of Internal Medicine, University of Medicine and Pharmacy of Craiova, 200349 Craiova, Romania; 4 Department of Pathology, University of Medicine and Pharmacy of Craiova, 200349 Craiova, Romania; mirela.florescu@umfcv.ro; 5 Department of Radiology and Medical Imaging, University of Medicine and Pharmacy of Craiova, 200349 Craiova, Romania; lucian.florescu@umfcv.ro; 6 Department of Rheumatology and Internal Medicine, Carol Davila University of Medicine and Pharmacy, 050474 Bucharest, Romania; anca.bobirca@umfcd.ro

**Keywords:** rheumatoid arthritis, interstitial lung disease, biomarkers, treatment

## Abstract

Rheumatoid arthritis (RA) is considered a systemic inflammatory disease marked by polyarthritis which affects the joints symmetrically, leading to progressive damage of the bone structure and eventually joint deformity. Lung involvement is the most prevalent extra-articular feature of RA, affecting 10–60% of patients with this disease. In this review, we aim to discuss the patterns of RA interstitial lung disease (ILD), the molecular mechanisms involved in the pathogenesis of ILD in RA, and also the therapeutic challenges in this particular extra-articular manifestation. The pathophysiology of RA-ILD has been linked to biomarkers such as anti-citrullinated protein antibodies (ACPAs), MUC5B mutation, Krebs von den Lungen 6 (KL-6), and other environmental factors such as smoking. Patients at the highest risk for RA-ILD and those most likely to advance will be identified using biomarkers. The hope is that finding biomarkers with good performance characteristics would help researchers better understand the pathophysiology of RA-ILD and, in turn, lead to the development of tailored therapeutics for this severe RA manifestation.

## 1. Introduction

Rheumatoid arthritis (RA) is considered a systemic inflammatory disease marked by polyarthritis, which affects the joints symmetrically, leading to progressive damage of the bone structure and eventually joint deformity. This pathology affects around 1% of the population in the United States and northern Europe [1,2]. Even though arthritis is the most prevalent clinical manifestation of RA, extra-articular manifestations are often evidenced in people with the disease. Extra-articular manifestations include cardiac, ocular, lung, cutaneous, gastrointestinal, neurological, and renal involvement, but also rheumatoid vasculitis and rheumatoid nodules [3,4].

Lung involvement is the most prevalent extra-articular feature of RA, affecting 10–60% of patients with this disease. Any segment of the respiratory tract can be affected in RA patients. The involved segments include the parenchyma, which can cause ILD or rheumatoid nodules, the pleura, causing pleural effusions or inflammation, the small and large airways (bronchiolitis, bronchiectasis, and cricoarytenoid inflammation), but also the pulmonary vessels, resulting in vasculitis and pulmonary hypertension. ILD is considered to have a prevalence ranging from 5 to 58%, clinically overt RA-ILD being encountered in less than 50% of patients [2,5,6]. 

Pleural effusion was thought to be the most frequent feature of RA-ILD before the development of computed tomography (CT), which aids in assessing the correct diagnosis. High-resolution computed tomography (HRCT) can identify more subtle changes in the parenchyma, leading to earlier discovery of the ILD, especially in subclinical phases when the patients have not developed symptoms such as dyspnea [7,8,9]. 

The aim of this review is to present the patterns involved in RA-ILD and the molecular mechanisms described in the pathogenesis of this extra-articular manifestation. We also aim to present the diagnostic and therapeutic approach in patients with RA-ILD. 

## 2. Pathogenesis

Rheumatoid factors (RF) and anti-citrullinated protein antibodies (ACPAs) are frequently found in the serum of RA patients. These autoantibodies are discovered in 50–80% of RA patients. They were discovered in the serum of patients with subclinical disease several years prior to clinical manifestations, thus testifying to the affirmation that genetic and environmental predispositions play an important part in the development of antibodies [10]. The production of antibodies leads to inflammation, followed by the development of clinical manifestations of the disease. Citrullination, the process through which arginine is converted to citrulline, leads to an immune response which implies the formation of ACPAs. ACPAs are significantly linked to the development of RA in those who are genetically susceptible [11,12]. 

Several immunopathogenic routes for RA-ILD have been proposed, although the precise location of the trigger event in the RA pathogenic cascade remains unknown. It is thought that the citrullinated proteins cross-react with the antigens in the lungs, albeit the immune response might be initiated in the synovium. This finding is reinforced by the fact that articular involvement precedes the pulmonary involvement in patients with RA. Recent literature data have shown that the microbiome plays a lead part in the development of RA due to its role in modulating the immune response. The “mucosal origins” theory posits that the development of RA begins in the mucosa of either the mouth, airway, or gastrointestinal tract. The bacterial, viral or mycobacterial antigens cross-react with antibodies, leading to the development of RA. Germs such as Proteus spp. and Porphyromonas gingivalis are thought to be involved in the pathogenesis of RA-ILD [13,14]. 

The genetic background of a patient might have either a predisposing (HLADRB1*15, HLADRB1*16, DQB1*06, and HLA-A*31:01 alleles) or protecting (HLA-DRB1 SE) role in the establishment of RA-ILD. Environmental conditions have a critical impact in genetically predisposed individuals. Tobacco usage has been identified as a probable cause of RA-ILD development. Smoking can harm pulmonary epithelial and vascular endothelial cells directly and increase citrullination of proteins in the lungs by activating PAD enzymes locally. Citrullinated proteins act as antigen targets, even in the preclinical stage, leading to a local immune response. This process leads to the formation of ACPAs, followed by the generation of RA and ILD. This stage is characterized by increased citrullination [15,16,17].

These formed antibodies lead to the development of an inflammatory response such as the production of pro-inflammatory cytokines, tumor necrosis factor (TNF)-α being one of the most important. B-cells are activated, and their differentiation is promoted by T-lymphocytes after antigen exposure. CD4^+^ T cells infiltrates are more prominent in RA-IL than CD3^+^ T lymphocytes, in contrast with idiopathic pulmonary fibrosis (IPF) infiltrates. Other researchers have speculated that CD8^+^ T cells might have a role in the progression of pulmonary fibrosis in RA. Certain data attest to the fact that CD8^+^ lymphocytes also have an important role in the development of ILD associated with RA, although this affirmation is portrayed in a study which suggests that smoking leads to an increase in CD8^+^ T lymphocytes in the lungs. [15,18,19,20].

The lung cellular infiltrates in RA-ILD have proven to be complex in SKG mouse models, consisting of CD4^+^ T lymphocytes, B lymphocytes, neutrophils, and macrophages. Cytokines and chemokines are of great importance in interstitial lung involvement in RA patients. TNF-α is a pro-inflammatory cytokine generated primarily by activated lymphocytes, macrophages, endothelial, and epithelial cells involved in the pathophysiology of ILD. TNF-α is important in the early stages and preservation of the cytokine and chemokine generation cascade and the induction of cell–cell adhesion and trans-endothelial migration [6,21].

The proliferation of fibroblasts is stimulated by TNF-α. Additionally, TNF-α promotes their capacity to degrade the extracellular matrix and to trigger the appearance of growth factors (GFs). GFs implied in the pathogenesis of ILD are platelet-derived growth factor (PDGF-β), transforming growth factor (TGF-β), but also vascular endothelial cell growth factor (VEGF). Nevertheless, the expression of cytokines such as IL-4 and IL-13, and chemokines (CXCL5, 8, 12, and 13) is also important. The GFs, cytokines and chemokines stimulate the fibroblasts to differentiate and proliferate, thus connecting the inflammatory and fibrotic stages. Macrophages, fibroblasts, epithelial, and endothelial cells all generate PDGF-β. PDGF-β is one of the pro-fibrotic, and pro-inflammatory molecules recognized to be important in the pathophysiology of ILD, such as TGF-β and TNF-α [5,22].

TGF-β’s profibrotic effect is mediated via monocyte and fibroblast recruitment and activation and the stimulation of extracellular matrix deposition. TGF-β also causes fibroblasts to differentiate into myofibroblasts, which are the primary source of the extracellular matrix in the process of fibrosis of the lungs. Chemokines do not have a well-defined role in the formation of the inflammatory infiltrates in the lungs of patients with RA-ILD. These chemokines are produced by macrophages, fibroblasts, and epithelial cells, and they function by recruiting and activating fibroblasts.

The pro-fibrotic and/or pro-inflammatory cytokines and GFs are known to activate the Janus kinase (JAK)/ signal transducer and activator of transcription (STAT) pathway. JAK/STAT activation leads to the polarization of macrophages into pro-inflammatory M1 type macrophages, with increases the secretion of cytokines such as IL-6, CXCL10 and TNF-α. These pro-inflammatory cytokines promote inflammation and/or fibrotic changes. 

**Figure 1:**
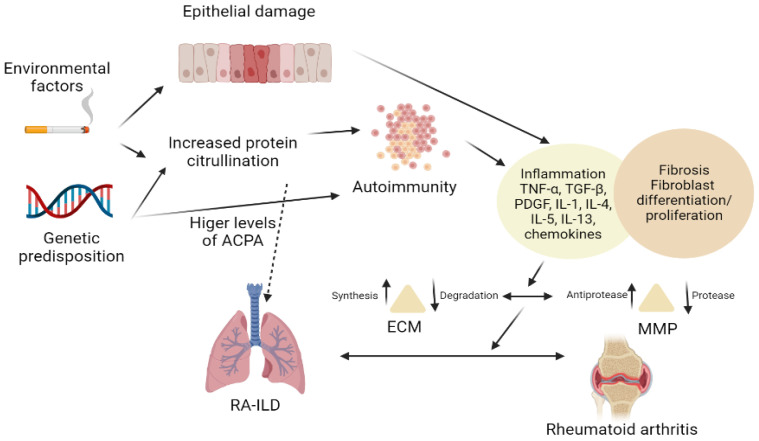
Pathogenesis of RA-ILD.

Other mediators included in the pathogenesis are matrix metalloproteinases (MMPs) produced by damaged epithelia. MMPs maintain the crosstalk between inflammation and fibrosis by increasing the recruitment of cells such as B and T lymphocytes, macrophages, and neutrophils and producing additional pro-fibrotic mediators. The inflammatory process promotes the generation of the VEGF which aids the angiogenetic process. The exact mechanism of the generation of VEGF is still not well determined (Figure 1).

## 3. Biomarkers

### 3.1. Antibody Biomarkers

Patients with RA are known to have a preclinical stage in which autoantibodies such as RF and/or ACPA are detected in the serum, before the appearance of clinical synovial inflammation. However, the presence of the serological markers in the serum in situations when ILD develops before the articular manifestations might somehow be confusing [23].

RF are autoantibodies oriented against modified Fc segments of immunoglobulin (Ig) G. RFs are found in the bloodstream of up to 80% of patients with RA. The majority of RF consists of IgM antibodies, which are linked to the development of interstitial lung involvement in RA. IgA RFs have also been linked to ILD [24].

ACPAs have specificity for proteins in which peptidyl arginine deaminases (PAD) have transformed the arginine residues and are seen in the sera of 70–80% of RA patients. Anti-PAD3 antibodies have been associated with interstitial lung disease. ACPAs are reported to have greater specificity for RA than RFs. ACPA levels over a certain threshold are linked to ILD in RA. It has been claimed that IPF is related to the generation of IgA type ACPAs, albeit this has not been linked to ILD as a RA consequence. Circulating secretory IgA-ACPAs have also been found in the serum of RA patients with ILD [25,26]. 

Other RA-ILD ACPAs have been discovered in RA-ILD patients. Antibodies against the citrullinated alpha-enolase peptide 1 (anti-CEP1) were linked to RA-ILD in an Italian investigation. In a Chinese study, it was also shown that increased levels of anti-CEP antibodies contributed to the development of ILD in RA patients. Antibodies against anticitrullinated heat shock protein 90 (cit-Hsp90) α or β have also been linked to RA-ILD, with low sensitivity but high specificity. Patients with RA-ILD produced more interferon g (IFN-γ) than those without ILD when their peripheral blood mononuclear cells (PBMC) were grown in contact with cit-Hsp90 beta. IFN- γ was not discovered in the PBMC of patients with other connective tissue diseases (CTD)-ILD. This is due to the fact that IFN- γ production is increased by cit-Hsp90 T lymphocytes specific for RA-ILD [19,27,28]. 

Antibodies targeting additional post-translationally modified proteins have been reported in addition to citrullinated proteins. Antibodies against anti-carbamylated proteins (anti-CarP) have recently been linked to the development of RA-ILD. Four anti-CarP antibodies were often discovered with high serum titers: IgG anti-fetal calf serum (FCS), antichimeric fibrin/filaggrin homocitrullinated peptide, anti-fibrinogen, and IgA anti-FCS. Finally, antibodies against malondialdehyde-acetaldehyde (anti-MAA) have been linked to lung involvement in RA. Anti-MAA antibodies have been linked with increased disease activity and response to ACPA [29,30,31].

Patients with RA-ILD had greater plasma levels of IgA and IgM anti-MAA antibodies than those with RA without ILD. Levels of IgM anti-MAA antibodies were also higher in patients with RA-ILD than in those with lung disease not related to RA, such as chronic obstructive pulmonary disease (COPD) [32,33].

To summarize, there is no evidence that ACPAs have a role in RA-ILD risk. In clinical practice, ACPA positivity should not be used as a predictor for the risk of development of RA-ILD. However, high anti-CCP antibody titers and rheumatoid factor titers may assist in identifying individuals with RA who are at high risk of ILD [34].

### 3.2. Genetic Biomarkers

Not many papers have reported the genetic connections involving interstitial lung involvement in RA, even though genetic risk factors for RA or IPF have been thoroughly researched. A single nucleotide variation (SNV) in the promoter region of the MUC5B gene, rs35705950, has been linked to familial and sporadic IPF. Additionally, a link has been established between RA-ILD and the mutation of the MUC5B gene [35,36].

The MUC5B gene is overexpressed when this risk allele is present. MUC5B overproduction may impede alveolar repair. On the other hand, this risk allele has been linked to a better prognosis in IPF patients, depicting its relevance in moderate IPF. In order to attest the influent aspect of common variations in disease predisposition, genome-wide association studies (GWASs) have been developed. In a Japanese GWAS, the SNV rs12702634 in the RPA3-UMAD1 gene proved to have a major association with the development of RA-ILD. This genetic polymorphism was mostly associated with the UIP pattern [37,38,39]. 

A study conducted by Jönsson et al. on 1466 RA patients from northern Sweden analyzed 571151 SNVs, finding that 4 of the tested SNPs were associated with interstitial lung involvement in RA, as follows: rs35705950 (MUC5B gene), rs2609255 (FAM13A), rs111521887 (TOLLIP gene), and rs2736100 (TERT gene). However, more extensive studies on a larger number of patients are yet to be conducted [40].

The antigens are provided to the T-cell receptors by HLA molecules; thus, HLA alleles are connected to a wide range of diseases. IPF is linked to HLA-B*15, HLA-B*40, HLA-DR2 (DRB1*15 and DRB1*16), and MICA*001. RA is linked to HLA-DRB1*04:01, *04:04, *04:05, *01:01, and *10:01. These RA risk alleles are known as “shared epitope” (SE) alleles because they share amino acid sequences at positions 70–74 of the HLA-DR protein (QKRAA, RRRAA, or QRRAA). In RA, DR2 alleles have been found to predispose to ILD, whilst SE alleles have been found to protect against ILD. Even though SE alleles are closely linked to ACPA-positive RA, the frequency of these alleles is lower in RA patients with interstitial lung involvement [41]. 

Micro RNAs (miRNAs) control the expression of genes that code proteins and are non-coding RNAs formed from about 22 nucleotides. Circulating miRNAs are rapidly emerging as disease biomarkers in several illnesses. Plasma levels of hsa-miR-214-5p and hsa-miR-7-5p are elevated in RA and IPF. Additionally, the potential of long non-coding RNAs has been tested. They are transcripts that are longer than 200 nucleotides, but do not have the capacity to be translated into proteins. The levels of several of these long non-coding RNAs were likewise shown to be higher in RA-ILD patients’ PBMCs [42,43].

### 3.3. Other Biomarkers

Krebs von den Lungen 6/MUC1 (KL-6) is a mucin-like glycoprotein which stimulates fibrosis and inhibits apoptosis of pulmonary fibroblasts. Serum KL-6 levels were shown to be higher in those with RA lung involvement, suggesting that it might help detect ILD development early on. In a study of 47 RA patients, the findings on lung computed tomography proved to be related to higher levels of serum KL-6 levels and increased disease severity. Severity was defined as extensive lung fibrosis on HRCT (>30%) or forced vital capacity (FVC) on PFT less than 50% and also the need of oxygen supplementation. Increased levels of KL-6 were also found in a study by Lee and colleagues in the serum of patients with CTD-ILD [44]. Type II pneumocytes and bronchiolar epithelial cells both express KL-6. KL-6 is expected to leak into the vascular system after epithelium breakdown caused by lung damage, indicating that it might be employed as a marker of epithelial injury. KL-6 might be used as a diagnostic marker in CTD-ILD. According to Oguz et al. in a study conducted on 113 CTD patients and 45 healthy controls, median KL-6 readings were significantly higher in the CTD-ILD group [44,45,46,47,48]. 

The pathophysiology of IPF is influenced by matrix metalloproteinases (MMPs) and tissue inhibitors of metalloproteinases (TIMPs), but also by cytokines and chemokines. MMP-7 levels were regularly observed to be higher in IPF patients. Several studies have looked at the involvement of these proteins in interstitial lung involvement in RA patients. High levels of MMP-7, soluble programmed death-ligand 1, C-X-C motif chemokine ligand 10 (CXCL10), interleukin (IL)-13, and IL-18 were discovered in the serum of patients with lung involvement in RA [49,50].

Chen et al. proved that MMP-7 and CXCL10 serum levels were more elevated in patients than those with RA without ILD [51]. Doyle et al. conducted a study which might help diagnose RA-ILD in the subclinical phase by discovering that a biomarker profile consisting of MMP-7, activation-regulation chemokines, and surfactant protein D (SP-D) is consistent with the development of ILD in RA patients [49,52,53].

Fu et al. discovered that lysyl oxidase-like 2 (LOXL2) levels in RA patients with or without ILD were higher in comparison with healthy controls. LOXL2 levels were substantially increased in subjects with RA-ILD who had ILD for ≤3 months than those who had ILD for >3 months [52]. The main candidates for biomarkers in RA-ILD are presented in Table 1.

In conclusion, each of these potential compounds, such as RF and ACPA, have some evidence of a link to RA-ILD. If any of these relationships are to be regarded as clinically effective biomarkers for RA-ILD, more research is needed to explain them and establish their validity. There are multiple ongoing clinical studies which aim to investigate biomarkers in RA-ILD, as presented in Table 2.

## 4. Similarities between RA-ILD and IPF

RA-ILD has certain phenotypic similarities with IPF, unlike other CTD-associated ILD. First, some risk variables are shared by RA-ILD and IPF, the most important being smoking, followed by age and male sex. On the second hand, they have a similar imaging and pathology phenotype, with an apparent prevalence of the usual interstitial pneumonia (UIP) pattern, which is the most prevalent pattern of interstitial lung involvement in RA [72]. 

ACPAs have recently been discovered in patients with IPF. ACPA positivity was shown to be more common in two different IPF cohorts. In these two IPF cohorts, IgA-ACPA positivity was higher than in the general population control group. The concept of a common genetic foundation in RA interstitial lung involvement and IPF is supported by phenotypic resemblance and shared environmental risk factors [73]. An increase in rare variations in genes associated with familial pulmonary fibrosis has been identified in RA-ILD. The functional MUC5B rs35705950 promoter mutation has recently been described as a risk factor for RA-ILD, in addition to being a significant risk factor for IPF. Strong MUC5B staining was evidenced in lung samples from individuals with RA-ILD, located in the areas with alveolar epithelium hyperplasia in the fibrotic regions, comparable to that seen in IPF. According to immunohistochemistry, IgA-ACPA positivity was higher than IgG-ACPA positivity in patients with IPF, whereas IgG-ACPA positivity was higher than IgA-ACPA positivity in patients with RA [74,75].

## 5. Diagnosis of RA-ILD

The diagnosis of ILD in patients with diagnosed or suspected RA demands a coordinated multidisciplinary approach involving radiology, pathology, rheumatology, and pulmonology expertise, as well as consideration of other possible causes of ILD. Each specialist has a well determined role in the diagnosis and treatment of ILD. After a diagnosis of RA is established by the rheumatologist, the patients have to be thoroughly evaluated. A HRCT has to be performed and interpreted by a specialized radiologist and if alterations in the lung parenchyma are detected, a complete evaluation with pulmonary function tests (PFTs) has to be conducted by a pulmonologist. Regarding the treatment, collaboration between the rheumatologist and pulmonologist is of great importance, since the therapeutic arsenal is different in each specialty. Thus, frequent meetings and conferences, or even the formation of multidisciplinary teams, are of great importance in the diagnosis and treatment of RA-ILD [76]. 

### 5.1. Clinical Presentation

Exertional dyspnea, cough, chest discomfort, and exhaustion are symptoms of ILD that are similar to those of a variety of more frequent lung disorders.

In individuals with fibrotic ILD, a clinical evaluation might reveal digital clubbing and/or Velcro-crackles on lung auscultation. Up to 15% of patients with RA-ILD have been reported to present clubbing [77,78].

Patients with RA-ILD have been found to exhibit bilateral basal crackles in almost 90% of cases. Crackles were found in individuals with RA who did not have ILD, albeit to a lesser level. The complexity of the illness and the diversity in HRCT patterns are most likely responsible for the clinical variability [34].

### 5.2. Imaging

The use of a chest X-ray to detect ILD in RA patients is ineffective. On a thoracic radiograph, up to 64% of individuals with ILD on HRCT will have no visible interstitial abnormalities. As a result, if ILD is suspected, HRCT must be performed as part of the diagnostic process.

The UIP pattern is the most frequently encountered in RA-ILD, although all types of interstitial pneumonia have been described. UIP, obliterative bronchiolitis, nonspecific interstitial pneumonia (NSIP), and organizing pneumonia (OP) were identified as the four primary HRCT patterns in individuals with RA-ILD [79,80]. 

### 5.3. Phenotypes of RA-ILD

The most prevalent type of ILD is usual interstitial pneumonia, evidenced in up to 70% of cases. It is associated with worse outcomes in comparison with other RA-ILD patterns. UIP typical HRCT features include a subpleural distribution with a basal predominance, honeycombing, which is highly specific, reflecting the stage and the severity of the disease, reticular opacities associated with honeycombing and traction bronchiectasis, ground-glass opacities, which are usually less extensive than the reticular pattern, architectural distortion, and lobar volume loss [81]. 

**Figure 2:**
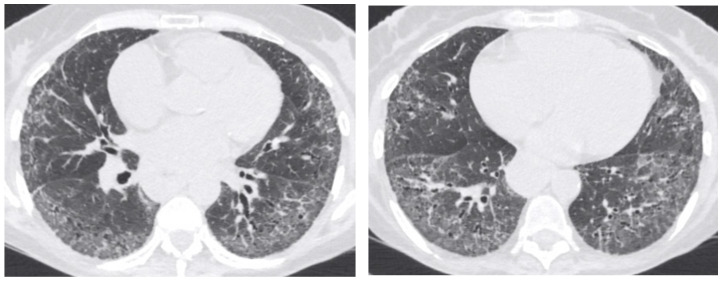
CT of the thorax—lung window—showing bilateral fine interstitial thickening and ground glass opacities with a basal predominance, minimal traction bronchiectasis and relative subpleural sparing (NSIP pattern).

Non-specific interstitial pneumonia is less prevalent than UIP. NSIP has two main subtypes: fibrotic and cellular, with lung involvement being mostly subpleural with an apicobasal gradient. NSIP typical HRCT features include ground-glass opacities with immediate subpleural sparing, mostly bilateral and symmetric, reticular opacities and irregular linear opacities, thickening of bronchovascular bundles, traction bronchiectasis, and lung volume loss, particularly in the lower lobes. It is associated with a lower risk of disease progression and a better response to treatment in comparison with UIP (Figure 2) [82]. 

Organizing pneumonia is a less frequent pattern encountered in RA-ILD. HRCT typical features include focal ground-glass opacities, consolidation and reversed halo sign. 

Other less common patterns are lymphocytic interstitial pneumonia (LIP) and desquamative interstitial pneumonia (DIP) [83]. 

LIP may present HRCT features such as diffuse with mid to lower lobe predominance, interstitial thickening along lymph channels, thickening of the bronchovascular bundles pulmonary nodules, either centrilobular or subpleural, ground-glass opacities, and thin wall cysts. 

DIP is characterized on HRCT by ground-glass opacities, irregular linear opacities, and small cystic spaces [84,85].

### 5.4. Pulmonary Function Tests

PFTs, especially the lung’s carbon monoxide diffusing capacity (DLCO), are able to detect subclinical pulmonary disease. The presence of concomitant emphysema and the variability of the PFTs within the normal values, may restrict the use of this diagnostic method. PFT outcomes in individuals with RA-ILD vary depending on the research groups and severity of the illness. PFT abnormalities are present in 45–65% of individuals with RA, whether or not they have respiratory symptoms.

Restrictive patterns, but also airway obstruction, and decreased DLCO are among the patterns. The incidence of a restricted pattern ranges from 5 to 25%. Approximately 20–45% of people with RA have a DLCO that is impaired. Although many people have abnormal PFTs, most of these abnormalities are clinically inconsequential and silent [86,87].

### 5.5. Bronchoalveolar Lavage

In individuals with RA-ILD, the cellular characteristics of bronchoalveolar lavage (BAL) fluid are frequently aberrant but nonspecific. Lymphocytosis tends to be more common in the non-UIP pattern, while increased neutrophil levels are characteristic of the UIP pattern. BAL is not always required. Usually, it is conducted to rule out other causes of lung disease. The nonspecific results prevent this method from being a useful diagnostic tool [88].

### 5.6. Histopathology

Insights into the histopathological structure of interstitial pneumonia obtained through surgical lung biopsy (SLB) may help to clarify the diagnosis and might also have prognostic significance. However, the risks outweigh the benefits in some cases, and the decision to perform a SLB needs to be carefully considered [89]. 

In RA-ILD, the histological patterns are varied, and any kind of interstitial pneumonia can occur and even overlap. Idiopathic interstitial pneumonias are classified according to a variety of distinct histological characteristics that are also observed in RA-ILD. Patches of fibrosis with honeycombing and fibroblast foci alternate with patches of normal lung tissue in a UIP pattern marked by heterogeneity. The appearance of NSIP is uniform, with thickening of the alveolar septa and various degrees of inflammatory and fibrotic changes. DIP and follicular bronchiolitis include peribronchiolar inflammation and fibrosis, while intra-alveolar connective tissue plugs characterize OP [90]. 

## 6. Treatment

It is critical to carry out a baseline evaluation of disease severity in patients diagnosed with RA-ILD and closely monitor patients to identify those who develop disease progression. When selecting whether to start or continue therapy in individuals with RA-ILD, the severity and progression of the illness are two significant variables to consider.

The best therapeutic plan for RA-ILD patients has yet to be determined. There have been no randomized controlled trials (RCTs) comparing drugs for the therapeutic options of RA-ILD to date [91].

### 6.1. Corticosteroids, Synthetic, Biological, and Targeted Therapy

In patients with refractory disease the most frequently utilized therapeutic strategies consist of corticosteroids, azathioprine, and mycophenolate, with rituximab or TNF-α inhibitors. In RA-ILD with an inflammatory pattern, treatment response is frequently better. Fibrotic lung disease, for example RA-UIP, is usually less responsive to treatment and disease progression is similar to IPF [92].

Current therapy is primarily centered on immunosuppression and is based on empirical information. Corticosteroids are usually administered either in a daily oral dose or as pulse therapy. The dose is tapered over several months according to tolerance and clinical response. In inflammatory types of RA-ILD, such as NSIP and OP, corticosteroids have proved to have a limited effect on disease progression. TNF-alpha inhibitors, methotrexate (MTX), azathioprine (AZA), mycophenolate mofetil (MMF), and cyclophosphamide (CYC) are among the immunosuppressive medications used as maintenance therapy or in corticosteroid-resistant cases [93].

Therapy with corticosteroids alone or in combination with DMARDs alleviated or stabilized the disease in almost half of the 84 patients with RA-UIP, according to a retrospective search by Song et al., but there was no substantial difference in lifespan compared to the untreated group [94].

In rapidly advancing, severe ILD and RA-ILD with substantial UIP, cyclophosphamide in conjunction with methylprednisolone have shown potential efficacy; however, the data is based on a limited retrospective case series [22].

In RA patients, methotrexate is recommended as the first-line therapy, since it successfully reduces disease progression, disability, and mortality. MTX, on the other hand, has been linked to the development or worsening ILD in RA patients [95]. Kiely et al. intended to see whether treatment with MTX is linked to RA-ILD diagnosis and delays RA-ILD development. They found that MTX exposure was linked with a substantially lower incidence of RA-ILD in a multicenter prospective early RA cohort analysis involving 2701 participants. Furthermore, they discovered that therapy may help RA patients postpone the onset of ILD. This research offers us reason to believe that MTX may be helpful in the prevention and treatment of RA-ILD [96].

In a study conducted by Yusof et al., rituximab (RTX) was administered in 700 individuals with RA, 56 of whom already had RA-ILD. After receiving rituximab, 68% of these patients had improved or maintained pulmonary function. Rituximab was shown to have a good safety profile, only three individuals (0.4%) having developed following therapy [96,97].

Interstitial lung involvement induced by medication has been cited for most TNF agents, including infliximab, adalimumab, etanercept, certolizumab pegol, golimumab, and IL-6 receptor antagonist tocilizumab. The majority of evidence for TNF inhibitors-related ILD comes from case reports. A thorough literature quest revealed that establishing a causal link between RA treatment and the beginning or progression of ILD is extremely challenging [98].

However, due to the lack of a dedicated RCT, the effect of bDMARDs on RA-ILD is uncertain. Rituximab, tocilizumab, and abatacept have all been shown to have favorable results in recent studies, with the disease in treated individuals maintaining constant or improving as measured by PFTs. However, most of these investigations are small, uncontrolled retrospective studies, and their findings must be confirmed in RCTs [99,100,101].

The JAK/STAT pathway is incriminated in the development of ILD. The beneficial effect of JAK inhibitors on CTD-ILD has been reported in a number of case reports presented in recent literature, in mouse models and in a few clinical studies. An open-label trial conducted by Chen Z. et al. evaluated the efficiency of tofacitinib in amyopathic dermatomyositis associated with ILD in patients with anti-melanoma differentiation-associated gene 5 (MDA5). The study involved 18 patients treated with GC and tofacitinib in doses of 10mg/day, while 32 patients treated with GC alone were included as historical controls. The 6 month survival rate was significantly higher in the group treated with tofacitinib than in the control group. Favorable outcomes were also noted in the case of FVC, DLCO and findings on the HRCT in the study group [102]. 

D’Alessandro et al. conducted a study on 15 patients (out of which 4 were diagnosed with RA-ILD) with RA in order to evaluate the adipokine levels in RA patients after 6 months of baricitinib treatment. The study showed a significant decrease in KL-6 levels in the patients with ILD, also showing an improvement in DLCO. Although the RA-ILD group was too small to have statistical significance, the results of this study may be a cornerstone for the development of other trials [103]. Other case reports on ruxolitinib have shown improvement in PFTs and HRCT in patients with ILD [104,105,106]. However, more expensive RCTs have to be conducted in order to establish the beneficial effect of JAK inhibitors in RA-ILD and the potential adverse events.

### 6.2. Antifibrotic Therapy

Due to the mechanistic similarities between RA-related UIP and IPF, antifibrotic medication may have a beneficial effect on progressive fibrotic RA-ILD, particularly with UIP patterns. Antifibrotic drugs are not known to be beneficial for articular symptoms of the condition, thus immunomodulating therapy may be needed in addition. When a varied group of patients with progressive fibrotic ILD (PF-ILD) (other than IPF) were placed together as a single entity, the results of the INBUILD study suggested a therapeutic advantage with nintedanib in individuals displaying pulmonary disease progression. A post hoc assessment of all diagnostic categories (including some autoimmune-ILDs) revealed a treatment advantage (particularly, the rate of FVC decline) [107,108,109,110].

Another treatment option is represented by pirfenidone which lowers serum concentrations of IL-6 and TNF-alpha, two important cytokines in RA pathogenesis. A recent discovery suggested that pirfenidone prevents the transition from fibroblast to myofibroblast in the lung tissues of patients with ILD. Due to this fact, treatment with pirfenidone may be considered in the case of UIP patterns. According to recent studies, pirfenidone has a beneficial effect on disease progression by slowing it in patients with unclassifiable PF-ILD [111,112].

The main limitation of our review is the fact that it is a narrative review, therefore eligibility criteria for studies, search strategy, selection process, study risk of bias assessment, and data collection are not explained.

## 7. Conclusions

For RA patients, ILD is a frequent and sometimes fatal consequence. Unfortunately, the precise etiology of RA-ILD is not fully understood yet. The pathophysiology of RA-ILD has been linked to biomarkers such as ACPA, MUC5B mutation, KL-6, and other environmental factors such as smoking. Patients at the highest risk for RA-ILD and those most likely to advance will be identified using biomarkers. The hope is that finding biomarkers with good performance characteristics would help researchers better understand the pathophysiology of RA-ILD and, in turn, lead to the development of tailored therapeutics for this severe RA manifestation. Although multiple biomarkers have been studied, none have proven performance characteristics in order to reliably identify interstitial lung disease in RA patients. More studies have to be performed in order to establish and validate the clinical implications, sensitivity, specificity, utility in diagnosis, prognosis and disease severity.

## Figures and Tables

**Table 1:** Value of biomarkers in RA-ILD.

Category	Biomarkers	Value	Evidence
Autoantibodies	ACPA	Diagnosis	High specificity—high titers associated with higher incidence of RA-ILD [54]
	Anticitrullinated HSP90	Diagnosis	Relatively high specificty and sensitivity [55]
	PAD	Severity	No utility for diagnosis—possible link to disease severity [56]
	Rheumatoid factor	Diagnosis	Low specificity—high titers associated with higher incidence of RA-ILD [57]
	Anti-CEP1	Diagnosis	More specific for synovial disease [58]
	Anti-CarP	Diagnosis	Relatively high specificity [59]
	Anti-MAA	Diagnosis	Not specific for RA-ILD—also found in RA without ILD [60]
Genetic biomarkers	MUC5B gene	Diagnosis	Highly suggestive, specifically in those with UIP pattern [61,62]
	microRNAs (has-miR-214-5p, has-miR-7-5p)	Diagnosis	High specificity, lower sensitivity—higher levels in patients with RA-ILD, not RA without ILD [63]
	HLA haplotypes (HLA-DR2, HLA-DQB1*04, *06, HLA-DR4, HLA-DRB1*14:06, HLA-DRB1*16:02-DQB1*05:02)	Diagnosis	Relatively low frequencies in RA-ILD [64]
Other biomarkers	KL-6	Severity	Highly suggestive for severity on HRCT [65,66]
	MMP-7	Diagnosis	Suggestive for fibrotic ILD—elevated in patients with ILD, not RA without ILD [41]
	CXCL10	Diagnosis	Elevated in patients with ILD, not RA without ILD [49]
	sPD-L1	Predictive	Relatively high specificity, lower sensitivity [67]
	IL-18	Diagnosis	Relatively high sensitivity and specificity [68]
	IL-13	Severity	Higher levels in RA-ILD, not RA without ILD [69]
	SP-D	Diagnosis	High specificity, lower sensitivity—influenced by bacterial lung infections [70]
	LOLX2	Diagnosis	High specificity for diagnosis [71]

ACPA—anticitrullinated protein antibodies; Anticitrullinated HSP90—heat shock protein 90; PAD—peptidyl arginine deaminases; anti-CEP1—citrullinated alpha-enolase peptide 1; anti-CarP—anti-carbamylated proteins; anti-MAA—anti- malondialdehyde-acetaldehyde; KL-6—Krebs von den Lungen 6/MUC1; MMP-7—matrix metalloproteinases 7; CXCL10—C-X-C motif chemokine ligand 10; sPD-1—soluble programmed death ligand 1; IL—interleukin; SP-D—surfactant protein D; LOLX2—lysyl oxidase-like 2.

**Table 2:** Clinical study in RA-ILD biomarkers.

Clinical Study	Type	Primary Outcome	Secondary Outcome	Biomarkers	Status/Results
Soluble Programmed Death 1 (sPD1) is a Diagnostic Biomarker of ILD in Patients With Rheumatoid Arthritis Disease NCT05105230 Year: 2021	Observational, Retrospective, Case-control, NP—66	Evaluation of the levels of serum (sPD1) in RA patients and its association with ILD	Detection of subclinical RA-ILD for early diagnosis and management of this extra-articular manifestation	sPD1	Not yet recruiting
Deciphering Rheumatoid Arthritis -associated Interstitial Lung Disease Pathogenesis 2 (TRANSLATE2) Year: 2020	Observational, Prospective, Case-control, NP—500	Identification of genetic factors involved in RA-ILD using whole exome sequencing (WES).	Identification of genetic factors implicated in RA-ILD using genome wide association study. Description of RA-ILD natural history by a 5 years annual follow up of RA-ILD patients. Description of the effect of disease modifying Anti Rheumatic Drugs (DMARDs) on ILD course and mortality.	Genetic	Recruiting
Rheumatoid Arthritis Patients at Risk for Interstitial Lung Disease (RAPID) NCT03297775 Year: 2017	Observational, Prospective, Cohort, NP—750	Presence or absence of ILD on HRCT imaging. The study is designed to evaluate individuals affected by RA and explore associated lung disease so that the investigators can better understand the clinical phenotype and genetic and molecular endotypes of this disease.		Multiple biomarkers (genetic, serum, sputum)	Recruiting
Lysophosphatidic Acid (LPA)/Autotaxin (ATX) Axis in Rheumatoid Lung Disease (LYSLUNG) NCT04284735 Year:2020	Interventional, Non-randomized NP—40	ATX and LPA levels in sputum from RA patients with ILD in comparison with sputum from RA patients without ILD	Correlation between ATX and LPA levels and severity of RA-ILD estimated by tomodensitometry	LPA/ATX	Recruiting
Rheumatoid Arthritis-Associated Interstitial Lung Disease: Characterization of Lung Disease Progression (BERTHA) NCT04136223 Year: 2019	Observational, Prospective, Cohort NP—100	Interstitial Lung Disease progression—FVC	Interstitial Lung Disease progression—imaging, death, FVC dichotomous variable	Genetic	Recruiting
Factors of ILD in Newly Diagnosed Rheumatoid Arthritis (FIND-RA) NCT04002765 Year: 2019	Interventional, Single group assignment NP—300	Presence of an interstitial lung disease	Proportion of different ILD subtypes Proportion of patients with a non-ILD lung involvement of RA rs35705950 variant of the MUC5B promoter Anti-CCP antibodies Relevant exposure	rs35705950 variant of the MUC5B promoter anti-CCP antibodies	Recruiting
Effects of Tofacitinib vs. Methotrexate on Clinical and Molecular Disease Activity Markers in Joints and Lungs in Early Rheumatoid Arthritis (PULMORA)—A Randomized, Controlled, Open-label, Assessor-blinded, Phase IV Trial NCT04311567 Year: 2020	Interventional, Randomized, Parallel assignment NP—48	Change in total interstitial disease score of pulmonary abnormalities by HRCT	Change in extent of parenchymal lung disease by HRCT pattern Change in Forced Vital Capacity (FVC) Change in Diffusion Capacity of Carbon Monoxide (DLCO) Change in walking distance (meters) Change in blood oxygen saturation (SpO2) after 6 min walking Patient reported outcome of breathing and airway symptoms	Exploratory sub-study: Gene expression of bulk tissue and sorted cells of synovial biopsies and broncho-alveolar lavage samples by RNA sequencing. Levels of cytokines (IL-1β, IFN-α2, IFN-γ, TNF-α, IL-6, IL-8 (CXCL8), IL-10, IL-12p70, IL-17A, IL-18, IL-23, and IL-33), chemokines (CCL2, CCL3, CCL4, CCL5, CCL11, CCL17, CCL20, CXCL1, CXCL5, CXCL8, CXCL9, CXCL10, CXCL11) and growth factors (GM-CSF, PDGF and TGFbeta1) of synovial fluid, blood and broncho-alveolar lavage will be determined by bead-based immunoassay.	Recruiting
Change in Serum and Sputum Biomarkers Over Time in the Development of Rheumatoid Arthritis-associated Lung Disease NCT03616158 Year: 2019	Observational, NP—340	Define which patients with RA (or pre-RA) are at greatest risk for developing RA-related lung disease (LD)		Multiple biomarkers (genetic, serum, sputum)	Recruiting

## Data Availability

Not applicable.
